# EZH2 mutations at diagnosis in follicular lymphoma: a promising biomarker to guide frontline treatment

**DOI:** 10.1186/s12885-022-10070-z

**Published:** 2022-09-14

**Authors:** C. Martínez-Laperche, L. Sanz-Villanueva, F. J. Díaz Crespo, P. Muñiz, R. Martín Rojas, D. Carbonell, M. Chicano, J. Suárez-González, J. Menárguez, M. Kwon, J. L. Diez Martín, I. Buño, M. Bastos Oreiro

**Affiliations:** 1 Gregorio Maranon Health Research Institute (IiSGM), Madrid, Spain; 2 Department of Hematology, Gregorio Marañón General University Hospital, Gregorio Marañón Health Research Institute (IiSGM), C/ Doctor Esuerdo 46, 28007 Madrid, Spain; 3 Pathology Department, Gregorio Maranon General University Hospital, Madrid, Spain; 4 Genomics Unit, Gregorio Maranon General University Hospital, IiSGM, Madrid, Spain; 5 Department of Medicine, School of Medicine, Complutense University of Madrid, Madrid, Spain; 6 Department of Cellular Biology, School of Medicine, Complutense University of Madrid, Madrid, Spain

**Keywords:** Follicular lymphoma, EZH2, R-Bendamustine, R-CHOP

## Abstract

*EZH2* is mutated in nearly 25% of follicular lymphoma (FL) cases. Little is known about how *EZH2* affects patients’ response to therapy. In this context, the aim of this study was to retrospectively analyze the frequency of mutations in *EZH2* at diagnosis in tissue and ctDNA in patients with FL and to assess the patients’ outcomes after receiving immunochemotherapy, depending on the *EZH2* mutation status. Among the 154 patients included in the study, 27% had mutated *EZH2* (46% with high-grade and 26% with low-grade FL). Of the mutated tissue samples, the mutation in ctDNA was identified in 44% of cases. *EZH2* mutation in ctDNA was not identified in any patient unmutated in the tissue.

Unmutated patients who received R-CHOP had significantly more relapses than patients who received R-Bendamustine (16/49 vs. 2/23, *p* = 0.040). Furthermore, our results show that patients with mutated *EZH2* treated with R-CHOP vs. those treated with R-Bendamustine present a lower incidence of relapse (10% vs. 42% *p* = 0.09 at 4 years), a higher PFS (92% vs. 40% *p* = 0.039 at 4 years), and higher OS (100% vs. 78% *p* = 0.039 at 4 years). Based on these data, RCHOP could be a more suitable regimen for mutated patients, and R-bendamustine for unmutated patients. These findings could mean the first-time identification of a useful biomarker to guide upfront therapy in FL.

**Supplementary Information:**

The online version contains supplementary material available at 10.1186/s12885-022-10070-z.

## Introduction

Follicular lymphoma (FL) is the second most common type of lymphoma diagnosed in Spain [1] and the United States [2], representing approximately 32% of all non-Hodgkin lymphomas (NHLs), and two thirds of indolent lymphomas. It is a germinal center origin disease, and nearly 90% of patients present translocation t(14;18) [3]. Chromatin modifying gene mutations (*KMT2D, CREBBP, EZH2*) are a common feature of FL [4]. It is characterized by an indolent course with a median overall survival (OS) beyond 10 years [5]. However, FL remains an incurable hematological malignancy with a characteristic course of multiple relapses, and with heterogeneous clinical behaviour, since about 20% of patients suffer from a rapid disease after treatment or a histological transformation to aggressive lymphoma (2% of patients per year) and a poor prognosis [6, 7].

The decision of therapy is strongly determined by the stage of the disease, the tumor burden, and the symptoms. In this sense, the most widely used tools for risk stratification, such as the Follicular Lymphoma International Prognostic Index (FLIPI) [8], and the PRIMA-prognostic index (PRIMA-PI) [9], m7-FLIPI [10], are not useful for selecting the best treatment strategy [11]. For localized disease, therapy options include radiotherapy [12], radiotherapy combined with immunochemotherapy [13], and observation without treatment, also known as watch & wait (W&W) strategy [14, 15]. Patients with advanced stage do not require immediate treatment, unless they have symptomatic or bulky, and are commonly observed under W&W strategy [16, 17] or may receive rituximab monotherapy [16, 18]. For advanced disease and high tumor burden, chemoimmunotherapy is the best option if GELF criteria are met [19, 20]. The most common chemoimmunotherapies used in combination with rituximab are cyclophosphamide, doxorubicin, vincristine, and prednisone (CHOP) [21], Bendamustine, or cyclophosphamide, vincristine, and prednisone (CVP) [22]. R-CHOP and R-Bendamustine (RB) have been compared in two different non-inferiority phase III clinical trials. Patients treated with RB presented a higher PFS but OS was similar to patients treated with R-CHOP [20, 23, 24]. Until today, the choice between one scheme or the other depends on the choice of the physician, or the centre’s protocols. Other anti-CD20 monoclonal antibodies, like Obinutuzumab, have been evaluated in combinations with Bendamustine or CHOP, and are also an option [25]. Patients with more aggressive lymphoma, such as histologically grade 3b or transformed, need to be treated as diffuse large B cell lymphoma with combinations including anthracyclines [20, 26].

In the past few years, next generation sequencing (NGS) has allowed us to approach the understanding of the genomic landscape and to discover more common mutations in FL, clarifying the lymphomagenesis mechanism, including epigenetic dysregulation [27]. Furthermore, this molecular approach has allowed for the establishment of a clinic-genetic risk model (m7-FLIPI), which includes the mutational status of seven genes [10]. This model includes the gene *EZH2*, which encodes the catalytic subunit of Polycomb repressor complex (PCR2) and mediates methylation of Lys27 residue of histone H3 (H3K27) [28, 29]. Missense mutations in *EZH2* lead to decreased transcriptional function of genes involved in cell cycle regulation and plasma cell differentiation, contributing to oncogenic transformation [4, 27, 28]. Mutated *EZH2* has been detected in nearly 25% of FL cases in tissue samples [30–34], but also could be analysed in cell tumor DNA (ctDNA) [35–38]. Although little is known about how *EZH2* affects patients’ response to therapy, Pastore *et* colleagues concluded that patients harboring *EZH2* were more likely to have better outcomes after most used R-CHOP [10]. Recently, in the 61st American Society of Hematology, Jurinovic et colleagues [39] considered *EZH2* one of the genes with higher impact in the m7-FLIPI when they assessed this risk model in patients treated with anti-CD20 combined with CHOP or bendamustine within the GALLIUM trial. These authors showed that patients who harbored mutated *EZH2* could benefit more from CHOP/CVP in combination with rituximab. These results also suggest that *EZH2* may have a predictive role in the selection of the chemoimmunotherapy for patients with FL.

In this context, the aim of this study was to retrospectively analyze, in a real-world setting, the frequency of mutations in *EZH2* at diagnosis in tissue and ctDNA in patients with FL and assess the patients’ outcomes with different upfront immune-chemotherapies, depending on the *EZH2* mutation status.

## Material and methods

### Patient samples

A total of 179 consecutive FL cases, diagnosed between 2002 and 2019 at the Department of Hematology, Gregorio Maranon General University Hospital, were included. Twenty-five out of 179 patients were excluded due to insufficient DNA quantity or quality or previous cancer. One hundred and fifty-four patients with histologically confirmed grade 1, 2, 3a or 3b FL, according to WHO classification and sufficient tissue available at diagnosis for DNA isolation, were eligible. Of the 154 patients analyzed, 39 had plasma samples at diagnosis or prior to treatment. Eligible patients were divided according to their grade of FL into low-grade FL (*n* = 141) (grades 1, 2 and 3a) and high-grade FL (*n* = 13) (grade 3b). Only high tumor burden patients that met criteria for treatment were included in the efficacy analysis. The study protocol was approved by the Ethical Committee of Gregorio Maranon General University Hospital (reference number HGM-EZH2-LF-2021) and all patients signed the informed consent document. All methods were performed in accordance with the relevant guidelines and regulations.

Clinical characteristics, therapy, and outcome were collected and shown in Tables 1 and 2 (grade 1, 2 and 3A) and 3 (grade 3B).

**Table 1:** Clinical characteristics, immunohistochemical and molecular markers available of patients with grade 1, 2 and 3A

	***N***	Total	Mutated ***EZH2*** in FFPE (***n*** = 36)	Unmutated ***EZH2***	***p-value***
		(***n*** = 141)		in FFPE (***n*** = 105)	
**Clinical characteristics at diagnosis, n (%)**					
Age at diagnosis, mean (range)	141	62 (15–90)	64 (42–90)	62 (15–89)	0.248
Sex					
Female	141	82 (58)	23 (64)	59 (56)	0.441
Male		59 (42)	13 (36)	46 (44)	
Histology					
Grade 1, 2	131	87 (66)	27 (77)	68 (72)	0.299
Grade 3A		44 (34)	8 (23)	26 (28)	
Ki67	124				
Low		55 (44)	16 (29)	39 (71)	0.54
Intermediate		47 (38)	12 (26)	35 (74)	0.84
High		22 (18)	5 (23)	17 (77)	0.45
Stage					
I-II	139	40 (29)	7 (21)	33 (33)	0.204
III-IV		99 (71)	28 (79)	71 (67)	
FLIPI risk categories					
Low-Intermediate	124	88 (71)	22 (65)	66 (73)	0.379
High		36 (29)	12 (35)	24 (27)	
Bulky mass	140	36 (26)	11 (31)	25 (24)	0.508
Extranodal	140	34 (24)	8 (22)	26 (25)	0.824
Bone narrow infiltration	139	47 (34)	13 (37)	34 (33)	0.682
B-symptoms	140	39 (28)	9 (25)	30 (29)	0.830

**Table 2:** Clinical characteristics, immunohistochemical and molecular markers available and response to therapy of patients with grade 3B

	***N***	Total	Mutated ***EZH2*** in FFPE (***n*** = 6)	Unmutated ***EZH2*** in FFPE (***n*** = 7)	***p-valor***
		(***n*** = 13)			
**Clinical characteristics at diagnosis, n (%)**					
Age at diagnosis, mean (range)	13	65 (30–85)	65 (42–84)	65 (30–85)	0.775
Sex					
Female	13	4 (31)	3 (50)	1 (14)	0.266
Male		9 (69)	3 (50)	6 (86)	
ECOG					
0–1	6	6 (100)	3 (100)	3 (100)	
Stage					
I-II	13	3 (23)	1 (17)	2 (29)	> 0.999
III-IV		10 (77)	5 (83)	5 (71)	
FLIPI risk categories					
Low-Intermediate	9	5 (56)	1 (20)	4 (100)	0.048
High		4 (44)	4 (80)	0	
Bulky mass	13	4 (31)	2 (33)	2 (29)	> 0.999
Extranodal	12	1 (8)	1 (20)	0	0.417
Bone narrow infiltration	13	4 (31)	3 (50)	1 (14)	0.266
B-symptoms	13	5 (39)	1 (17)	4 (57)	0.266
**First-line R-CHOP therapy, n (%)**	13				
Complete remission		8 (62)	3 (60)	5 (71)	0.54
Progression		0			NA
Relapse		3 (23)	2 (33)	1 (14)	0.2
Not assessed^a^		2 (15)	1 (17)	1 (14)	
**Exitus, n (%)**					
Yes	13	4 (31)	1 (17)	3 (43)	0.559
No		9 (69)	5 (83)	4 (57)	

^a^Data not included in Fisher’s exact test

### Genetic analysis

DNA was extracted from tissue biopsies using Maxwell(R) 16 FFPE Plus LEV DNA Purification Kit (Promega) or GeneRead DNA FFPE Kit (Qiagen). Cell free DNA was isolated from plasma samples using QIAamp® Circulating Nucleic Acid (Qiagen). Mutations in DNA tissue was performed by Sanger sequencing (ABI3130xl DNA sequencer) (*n* = 139) (Supplementary Table 1) and 15 patients using a capture-based targeted commercial panel of 54 genes (Lymphoma Solution, Sophia Genetics; Next Seq, Illumina) [40]. Bioinformatics analyses were performed using DDM software (Sophia Genetics). Mutations in ctDNA was performed by RT-qPCR reactions on a Roche Light Cycler 480 Instrument II Real Time PCR System, using Prime Time Mini LNA probes for mutations Y646N, Y646S, Y646C, and A692V (57% of existing variants) (Supplementary Tables 2–4).

### Statistical analysis

Data analysis, including descriptive statistics and Fisher’s exact test, was performed using IBM SPSS Statistics 26 (IBM, USA). OS was defined as the time from diagnosis (date of biopsy) to death or last visit if the patient was still alive. PFS was defined as the time from treatment onset to progression or last visit if there was no progression. Both OS and PFS of the total cohort, and according to therapy received and *EZH2* mutation status, were calculated using R studio Version 1.3.1056 (RStudio, Inc.) Progression of disease within 24 months (POD24) was defined from diagnosis to progression if occurring within 24 months. *P*-values of less than 0.05 were considered significant.

## Results

### Distribution of EZH2 mutations at diagnosis

- *Global cohort*

Of the total cohort (*n* = 154), in tissue, 42 (27%) presented *EZH2* mutations at diagnosis. Mutations are missense and were detected at 3 recurrent mutation hot spots (Y646, A682, and A692). The most frequent mutation was Y646N (18, 42%), followed by Y646F (9, 21%), A682G (5, 11.9%), A692V (4, 9.5%), Y646C (3, 7.1%), Y646H (2, 4,7%), and Y646S (1, 2.3%).

Patients were then divided into two groups according to their grade of FL: low-grade FL (grades 1, 2 and 3a) and high-grade FL (grade 3b).

- *High-grade FL*

*EZH2* mutations were found in 6 of the 13 (46%) high-grade FL patients’ tissue (Table 3). Mutations detected were Y646N (2.3%), Y646F (1.2%), A682G (1.2%), Y646C (2.2%), and Y646S (1.2%). Among patients with mutated *EZH2*, 2 of those with advanced disease had plasma available. The same mutation as detected in tissue (Y646N) was also identified in ctDNA.

- *Low-grade FL*

**Table 3:** Response to therapy of patients with grade 1, 2 and 3A

	***N***	Total	Mutated ***EZH2*** in FFPE (***n*** = 36)	Unmutated ***EZH2*** in FFPE (***n*** = 105)	***p-valor***
		(***n*** = 141)			
**Outcome, n (%)**					
**First-line therapy, n (%)**	**141**				
Treated		123 (87)	30 (83)	93 (89)	0.4
Watchful waiting		18 (13)	6 (17)	12 (11)	
Progression		4 (3)	1 (2.8)	3 (2.9)	0.7
Transformation		4 (3)	1 (2.8)	3 (2.9)	0.7
POD24		13 (9.2)	4 (11.1)	9 (8.6)	0.4
Relapse		25 (17.8)	4 (11)	21 (20)	0.1
Death		22 (15.6)	2 (5.5)	20 (19)	0.034
*R-Bendamustine*	**30**	**30 (24)**	**7 (23)**	**23 (25)**	
Complete remission		29 (97)	7	22 (96)	0.8
Partial remission		0	0	0	NA
Progression		1 (3.5)	0	1 (4.4)	NA
Transformation		0	0	0	NA
POD24		4 (13.3)	2 (29)	2 (9)	0.2
**Relapse**		4 (13.3)	2 (5.6)	2 (1.9)	0.2
Death		4 (13.3)	1 (14.2)	3 (13)	0.7
*R-CHOP*	**67**	**67 (55)**	**18 (60)**	**49 (52)**	
Complete remission		62 (93)	16 (89)	48 (98)	0.8
Partial remission		1 (1.5)	0	1 (2)	NA
Not assessed^a^		2 (3)	2 (11)	0	NA
Progression		0	0	0	NA
Transformation		0	0	0	NA
POD24		6 (9)	1 (5.5)	5 (10)	0.4
**Relapse**		**18**	**2 (11)**	**16 (32)**	0.052
Death		11 (16.5)	1 (2.8)	10 (9.5)	0.13
*R-CVP, Rituximab and Radiotherapy*	**26**	**26 (21)**	**5 (17)**	**21 (23)**	

^a^Data not included in Fisher’s exact test

*EZH2* mutations were detected in 36 out of 141 (26%) low-grade FL patients’ tissue (Tables 1 and 2). The most frequent mutation was Y646N (16.4%), followed by Y646F (8.2%), A682G (5.1%), A692V (2.6%), Y646C (2.6%), Y646H (2.6%), and Y646S (1.3%). Of the 37 plasma samples collected, 4 cases were positive in ctDNA (2 patients presented Y646N, 1 Y646S, and 1 Y646C), all of them were also present in tissue, 3/ 4 patients (75%) had advanced stage. Five ctDNA samples were *EZH2* negative and positive in tissue. Therefore, 44% of mutated patients with available ctDNA at diagnosis had the mutation in ctDNA. We did not identify *EZH2* mutations in ctDNA from patients with unmutated *EZH2* in tissue.

### Clinical correlations and prognostic value of EZH2 mutation

Clinical and biological characteristics and outcome were compared in low-grade FL (Tables 1 and 2) and high-grade FL (Table 3) according to *EZH2* mutation status in tissue.

#### High-grade FL

In high-grade FL, mutated *EZH2* had statistically higher FLIPI risk (100% vs. 0%; *p* = 0.048). No statistical differences were found when comparing the resting clinical and biological characteristics and outcome in mutated and unmutated patients. All patients included in the high-grade FL group were treated with R-CHOP (13) (Table 2).

#### Low-grade FL

In low-grade FL, there was no statistical significance when comparing clinical characteristic immunohistochemical and molecular markers between mutated and unmutated *EZH2* in tissue (Table 1). One hundred and twenty-three patients received treatment and 18 were observed according to the W&W strategy. Of the total of treated patients, 30 received R-Bendamustine, 67 R-CHOP, and 26 R-CVP, Rituximab or radiotherapy (Table 3). Patients with EZH2 mutated vs. no present lower relapses (11% VS. 20% *p* = 0.1), with no statistical differences and lower deaths (5.5% vs. 19% *p* = 0.034) Table 3.

**Fig. 1:**
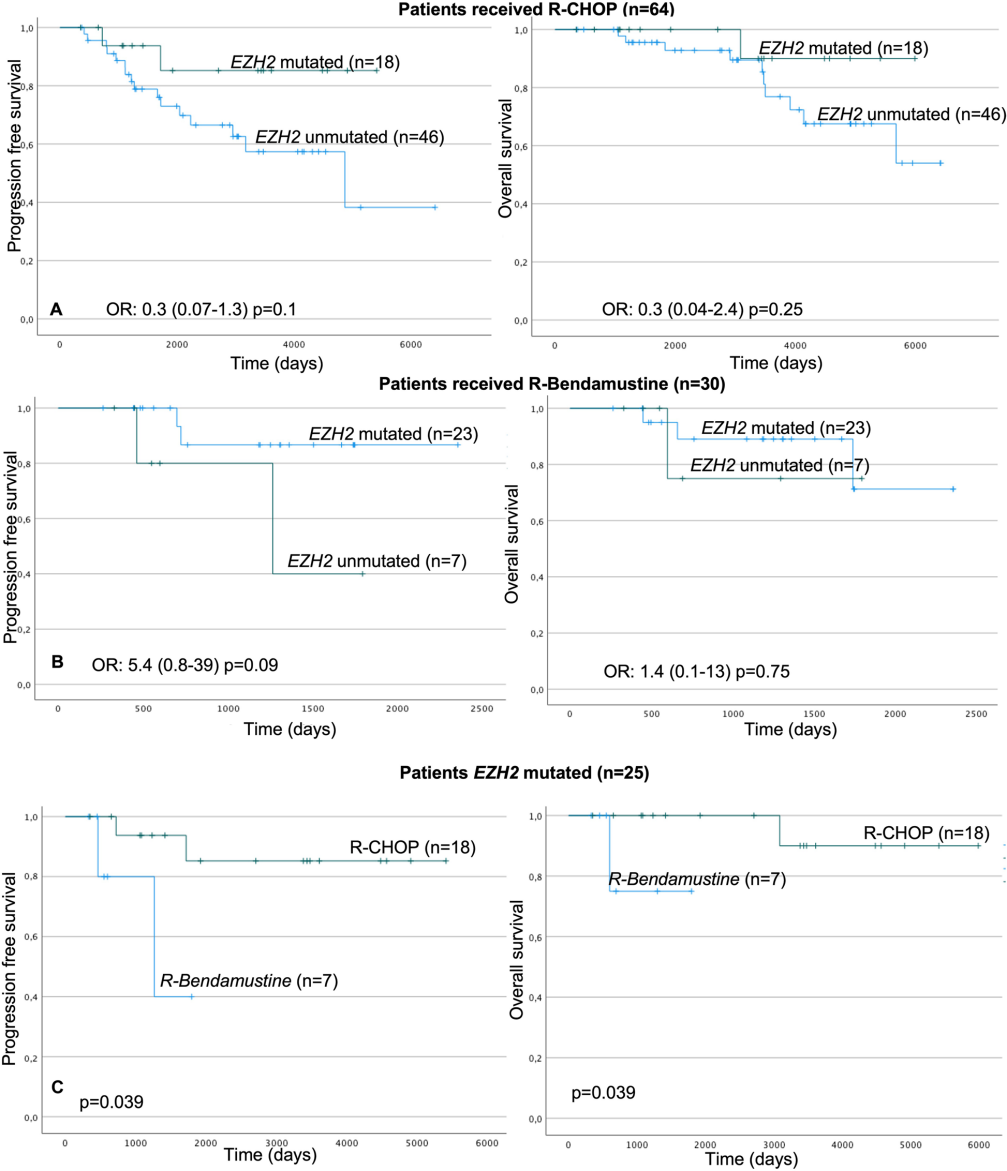
Kaplan-Meier curves in patients with grade 1, 2, and 3a. **A** PFS and OS in patients treated with R-CHOP (*EZH2* mutated vs. unmutated); **B** PFS and OS in patients treated with R-Bendamustine (*EZH2* mutated vs. unmutated). **C** PFS and OS in *EZH2* mutated patients (R-CHOP vs. R-Bendamustine). PFS Progression-free survival. OS: Overall survival

Outcomes after first line therapy in mutated and unmutated patients were compared between R-Bendamustine and R-CHOP. Clinical and biological characteristics between the groups (R-CHOP vs. R-Bendamustine) were analysed with no significant differences found between them (Supplementary Table 5). Percentage of patients that achieved complete remission after receiving R-Bendamustine or R-CHOP were similar when compared according to *EZH2* status. There were no statistical differences when comparing POD24 according to therapy received and *EZH2* mutational status. Instead, unmutated patients who received R-CHOP had significantly more relapses than patients who received R-Bendamustine (16/49 vs. 2/23, *p* = 0.040), no differences were found in terms of PFS and OS (data not shown). Furthermore, our results show that mutated *EZH2* patients treated with R-CHOP vs. those treated with R-Bendamustine present a higher PFS (92% vs. 40% at 4 years *p* = 0.039), and higher OS (100% vs. 78% at 4 years *p* = 0.039), Fig. 1C.

If the analysis is carried out regarding the treatment received, the group of patients who received R-CHOP, mutated vs. unmutated *EZH2* patient’s trend to present a higher PFS (at 4 years 92% vs.79% *p* = 0.1) and a higher OS (at 11 years 90% vs. 70% *p* = 0.2) (Fig. 1A). In the group of patients who received R-bendamustine, mutated vs. unmutated *EZH2* patient’s seem to have a lower PFS with no statistical differences (at 4 years 40% vs.85% *p* = 0.09). No differences were found in terms of OS (Fig. 1B).

## Discussion

In this study, we evaluate the frequency of *EZH2* mutations in tissue biopsy and in ctDNA at the time of diagnosis in FL, and we describe the possible usefulness of this biomarker as a tool to guide frontline treatment. We have found 27% of patients mutated in low-grade FL and 46% in high-grade. The frequency in which we have found mutated low-grade LF patients is similar to that previously reported [41, 42]. However, 46% of patients were mutated in the high-grade FL group. This remarkable difference between low and high-grade FL has not been previously referred to, as far as we know. In fact, looking at the percentage of mutated patients in DLCBL, even those of germinal center origin, it is significantly lower, near 20% [32, 43]. It is important to note that our population of patients with high-grade FL is small, and we must increase it in order to confirm these data.

Furthermore, here we showed that *EZH2* mutations are detectable in ctDNA. In the present study we found that 44% of mutated patients with available ctDNA at diagnosis had the mutation. The percentage of ctDNA with mutated *EZH2* is probably misrepresented, because in some samples the amount of plasma obtained was low and, furthermore, a higher number of samples in low-grade FL could be needed since the amount of ctDNA released into the plasma in this group may be lower than in high-grade lymphomas [40]. It should be noted that 83% of the ctDNA with mutated *EZH2* in our cohort had stage III-IV. It seems to be more common to find the ctDNA that harbors mutations in this gene in advanced stage patients, probably because in this group a greater amount of ctDNA is being released into the bloodstream. In this regard, the quantification of ctDNA has been directly related to the tumor metabolic volume in FL [36].

In high-grade FL, mutated *EZH2* had statistically higher FLIPI risk (100% vs. 0%; *p* = 0.048). No statistical differences were found when comparing the resting clinical and biological characteristics and outcome in mutated and unmutated patients. In low-grade FL, there was no statistical significance when comparing clinical characteristic immunohistochemical and molecular markers between mutated and unmutated *EZH2* in tissue.

As previously mentioned, the decision about first-line treatment in advanced stage low-grade FL is difficult, and so far we do not have prognostic tools that allow the selection of one immunochemotherapy regime over the other [44]. *EZH2* has been highlighted as a prognosis predictor [10, 38, 39]. A study that evaluates the impact of genetic alterations in *EZH2* in a group of patients treated homogeneously with RCHOP within a clinical trial identified those patients with alterations as having longer PFS compared to those who did not have them [31]. Likewise, in a recent international hematology meeting it was postulated as a tool for frontline therapy selection in patients with low-grade FL, results similar to ours [39]. In this abstract the author postulated that *EZH2* mutation status was associated with longer PFS in patients receiving CHOP/CVP regimens and it did not impact treatment outcome of patients treated with R-Bendamustine, suggesting that *EZH2* mutation status could be a predictive marker for differential efficacy of the chemotherapy regimen. In our study we have found that patients with *EZH2* mutated low-grade FL treated with R-CHOP had significantly lower incidence of relapse, and higher PFS and OS compared to those treated with R-Bendamustine. To our knowledge, this is the first time that this correlation between the *EZH2* mutation and the type of immunochemotherapy used in the first-line treatment of FL has been published.

*EZH2* mutation occurs early in the development of FL [28]. However, it has been postulated as one of genes that promotes progression, relapse or transformation in FL, since some patients acquired it during this event [42, 45–47]. Taking into account that in our high-grade patients the mutation is significantly more represented, and the best PFS obtained with the use of anthracyclines in the mutated group could be an indicator of high-grade comportment of the disease, although other studies would be necessary to demonstrate this hypothesis, furthermore the number of patients with high grade FL (*n* = 13) is low, we must confirm this data in a higher cohort.

The present study has several limitations. The first being the one inherent to the retrospective nature of the study. On the other hand, there could be a selection bias when selecting the treatment, since in routine clinical practice, in patients who have clinical behaviour of transformation, although the biopsy does not confirm it, these could tend to be treated more with RCHOP instead of R-Bendamustine. Likewise, the expression and copy numbers of *EZH2* have not been studied, which could enrich the results, but would require a different technology than that used in our analysis. Finally, the number of patients treated in first line with R-CHOP and R-Bendamustine must be expanded to confirm our results. Despite this, we consider that our findings are of great importance and may have an impact on the therapeutic decision of patients with LF in the first line. In conclusion, our study shows that the status of the *EZH2* mutation in FL at diagnosis could be a useful marker for the selection of first-line treatment in low-grade FL. Furthermore, ctDNA could be a promising tool in identifying mutated patients, especially in advanced stages and high-grade FL.

## Supplementary Information

**Additional file 1: Supplementary Table 1.** Custom set of primers for mutations in exons 16 and 18 for sanger sequencing. **Supplementary Table 2.** Custom set of probes and primers for mutation Y646N for RT-qPCR. **Supplementary Table 3.** Custom set of probes and LNA primers for mutations Y646C and Y646S for RT-qPCR. **Supplementary Table 4.** Custom set of probes and LNA primers for mutation A692V for RT-qPCR. **Supplementary Table 5.** Clinical characteristics, immunohistochemical and molecular markers available in patients treated with R-Benda VS. R-CHOP.

## Data Availability

The datasets used and/or analysed during the current study are available from the corresponding author on reasonable request.
