# Correction: EZH2 mutations at diagnosis in follicular lymphoma: a promising biomarker to guide frontline treatment

**DOI:** 10.1186/s12885-022-10335-7

**Published:** 2022-11-28

**Authors:** C. Martínez-Laperche, L. Sanz-Villanueva, F. J. Díaz Crespo, P. Muñiz, R. Martín Rojas, D. Carbonell, M. Chicano, J. Suárez-González, J. Menárguez, M. Kwon, J. L. Diez Martín, I. Buño, M. Bastos Oreiro

**Affiliations:** 1 Gregorio Maranon Health Research Institute (IiSGM), Madrid, Spain; 2 Department of Hematology, Gregorio Marañón General University Hospital, Gregorio Marañón Health Research Institute (IiSGM), C/ Doctor Esuerdo 46, 28007 Madrid, Spain; 3 Pathology Department, Gregorio Maranon General University Hospital, Madrid, Spain; 4 Genomics Unit, Gregorio Maranon General University Hospital, IiSGM, Madrid, Spain; 5 Department of Medicine, School of Medicine, Complutense University of Madrid, Madrid, Spain; 6 Department of Cellular Biology, School of Medicine, Complutense University of Madrid, Madrid, Spain

**Correction: BMC Cancer 22, 982 (2022)**

**https://doi.org/10.1186/s12885-022-10070-z**

Following publication of the original article [1], the authors identified an error in Fig. 1B. Patients with EZH2 mutation (n=7) have worse prognosis than patients without mutation (n=23). The updated Fig. 1 is supplied in this correction article.

**Fig. 1:**
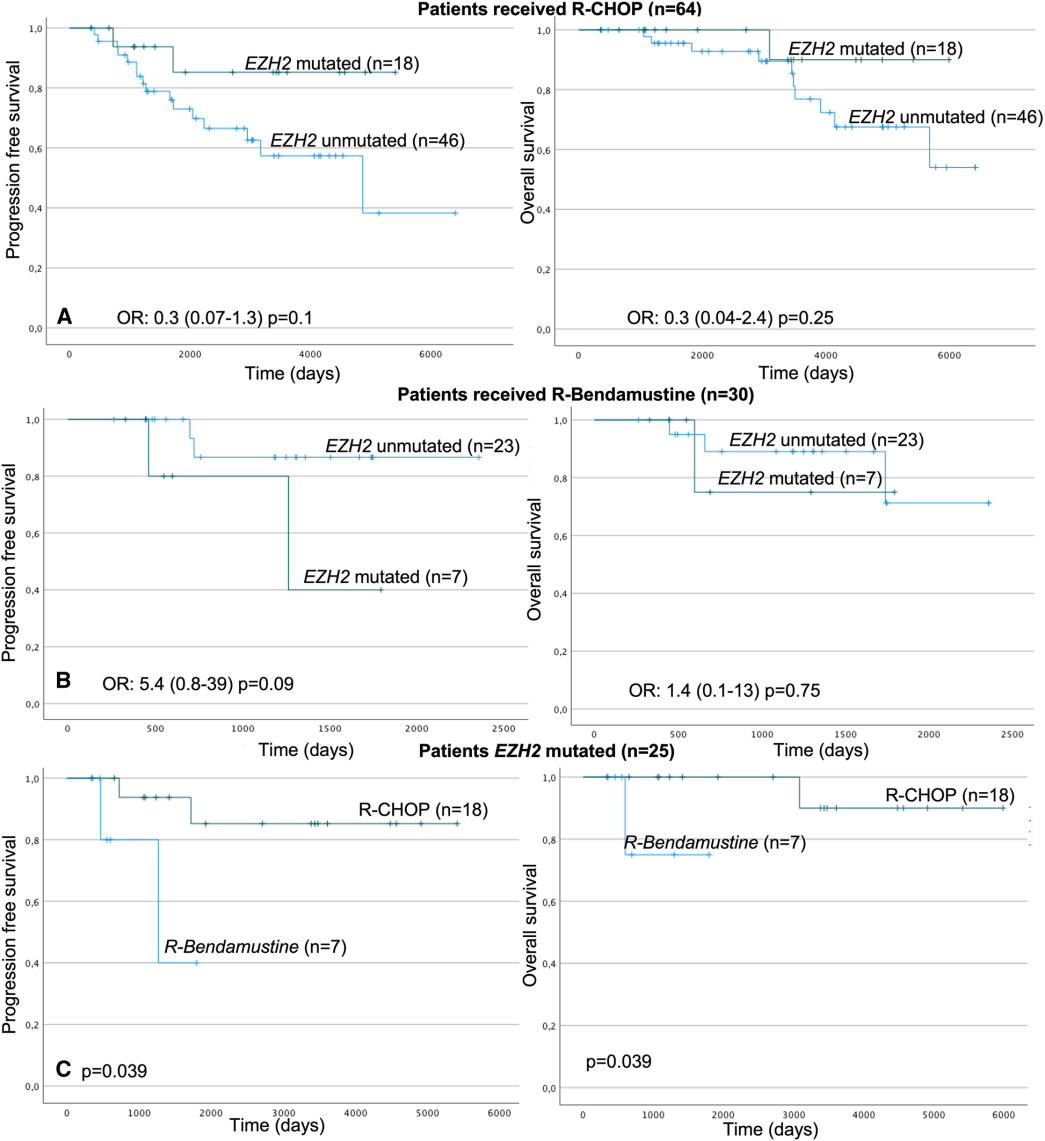
Kaplan-Meier curves in patients with grade 1, 2, and 3a. **A** PFS and OS in patients treated with R-CHOP (*EZH2* mutated vs. unmutated); **B** PFS and OS in patients treated with R-Bendamustine (*EZH2* mutated vs. unmutated). **C** PFS and OS in *EZH2* mutated patients (R-CHOP vs. R-Bendamustine). PFS Progression-free survival. OS: Overall survival

The original article [1] has been corrected.
