# Supplementary material for: EZH2 mutations at diagnosis in follicular lymphoma: a promising biomarker to guide frontline treatment
Source: BMC Cancer. 2022 Sep 14;22:982. doi: 10.1186/s12885-022-10070-z (PMC9476261; doi:10.1186/s12885-022-10070-z)
Supplement: Supplementary file 1 — Additional file 1: Supplementary Table 1. Custom set of primers for mutations in exons 16 and 18 for sanger sequencing. Supplementary Table 2. Custom set of probes and primers for mutation Y646N for RT-qPCR. Supplementary Table 3. Custom set of probes and LNA primers for mutations Y646C and Y646S for RT-qPCR. Supplementary Table 4. Custom set of probes and LNA primers for mutation A692V for RT-qPCR. Supplementary Table 5. Clinical characteristics, immunohistochemical and molecular markers available in patients treated with R-Benda VS. R-CHOP. [file 12885_2022_10070_MOESM1_ESM.docx]

Supplementary information

Supplementary Tables

| **Supplementary table 1.** Custom set of primers for mutations in exons 16 and 18 for sanger sequencing | |
| --- | --- |
| **Exon 16** | **Sequence** |
| Forward | 5'-GTG TGC CCA ATT ACT GCC TT-3' |
| Reverse | 5'-CAT TTC CAA TCA AAC CCA CA-3' |
| **Exon 18** | **Sequence** |
| Forward | 5'-AGG CAA ACC CTG AAG AAC TG-3' |
| Reverse | 5'-CAC ACT GGT GTC AGT GAG CAT-3' |
|  |  |
| **Supplementary table 2.** Custom set of probes and primers for mutation Y646N for RT-qPCR | |
| **Probes** | **Sequence** |
| *EZH2^Y646N^* | /56-FAM/AGA A+TA CTG +TGG A+GA GGT A/3IABkFQ/^1^ |
| *EZH2^WT^* | /56-FAM/AGA A+TA CTG +TGG A+GA GGT A/3IABkFQ/^1^ |
| **Primers** | **Sequence** |
| Forward | 5'-TGA ATA CAG GTT ATC AGT GC-3' |
| Reverse | 5'-TCA AAG ATC CTG TGC AGA-3' |
|  |  |
| **Supplementary Table 3.** Custom set of probe and LNA primers for mutations Y646C and Y646S for RT-qPCR | |
| **Probe** | **Sequence** |
| *EZH2^Y646^* | /56-FAM/AGC ATC TAT /ZEN/TGC TGG CAC CAT CT/3IABkFQ/ |
| **Primers** | **Sequence** |
| *EZH2^Y646C^* | 5'-GCC TTA CCT CTC CAC AG+C-3' |
| *EZH2^Y646S^* | 5'-GCC TTA CCT CTC CAC AG+G-3' |
| *EZH2^WT^* | 5'-GCC TTA CCT CTC CAC AGT AT-3' |
| Common forward | 5'-CTG TAG TCT ACT TTG TCC CC-3' |
|  |  |
| **Supplementary Table 4.** Custom set of probe and LNA primers for mutation A692V for RT-qPCR | |
| **Probe** | **Sequence** |
| *EZH2^A692V^* | /5HEX/AGG TAG GTA /ZEN/CCT TTG ACG TGA /3IABkFQ/ |
| **Primers** | **Sequence** |
| *EZH2^A692V^* | 5'-GGG TAA CAA AAT TCG TTT TGT T-3' |
| *EZH2^WT^* | 5'-GGG TAA CAA AAT TCG TTT TGC A-3' |
| Common reverse | 5'-CAC ACT GGT GTC AGT GAG CAT-3' |

1. Alcaide, M. *et al.* Multiplex droplet digital PCR quantification of recurrent somatic mutations in diffuse large b-cell and follicular lymphoma. *Clin. Chem.* **62**, 1238–1247 (2016).

**Supplementary Table 5.** Clinical characteristics, immunohistochemical, molecular markers available in patients treated with R-Benda VS. R-CHOP.

|  | | | | |
| --- | --- | --- | --- | --- |
|  | ***N*** | **Treated**  **R-CHOP (n=67)** | **Treated**  **R-Benda (n=30)** | ***p-value*** |
| **Clinical characteristics at diagnosis, n (%)** |  |  |  |  |
| Age at diagnosis, mean (range) |  | 57 (15-84) | 62 (36-82) | 0.4 |
| Sex | 97 |  |  |  |
| Female |  | 38 (56.7) | 15 (50) | 0.5 |
| Male |  | 29 (43.2) | 15 (50) |  |
| ECOG | 74 |  |  |  |
| 0-1 |  | 46 (97.8) | 25 (92.5) | 0.3 |
| ≥2 |  | 1 (2.1) | 5 (7.4) |  |
| Histology | 97 |  |  |  |
| Grade 1, 2 |  | 45 (67) | 25 (83) | 0.08 |
| Grade 3A |  | 22 (33) | 5 (17) |  |
| Stage | 96 |  |  |  |
| I-II |  | 10 (15.1) | 8 (26.7) | 0.18 |
| III-IV |  | 56 (84.8) | 22 (73.4) |  |
| Immunohistology |  |  |  |  |
| BCL2 | 91 | 57/62 (91.9) | 26/29 (89.6) | 0.7 |
| BCL6 | 87 | 56/59 (94.9) | 27/28 (96.4) | 0.7 |
| CD10 | 88 | 48/61 (78.6) | 25/27 (92.6) | 0.1 |
| CD20 | 92 | 62/62 (100) | 29/30 (96.7) | 0.15 |
| FISH |  |  |  |  |
| Positive BCL2 rearragement | 59 | 16/26 (61.5) | 16/33 (48.5) | 0.3 |
| Positive BCL6 rearragement | 54 | 01/24 (4.2) | 7/30 (23.3) | 0.049 |
| EZH2 mutated | 97 | 18 (26.8) | 7 (25) | 0.7 |
| FLIPI risk categories | 85 |  |  |  |
| Low-Intermediate | 56 | 34 (60.7) | 22 (75.8) | 0.16 |
| High | 29 | 22 (39.3) | 7 (24,1) |  |
| Bulky mass | 96 | 24 (36.3) | 10 (33.4) | 0.7 |
| Extranodal | 96 | 16 (24.2) | 10 (33.3) | 0.3 |
| Bone marrow infiltration | 96 | 27 (40.9) | 12 (40) | 0.9 |
